# Changes in Bone Metabolism and Antioxidant Defense Systems in Menopause-Induced Rats Fed Bran Extract from Dark Purple Rice (*Oryza sativa* L. Cv. Superjami)

**DOI:** 10.3390/nu13092926

**Published:** 2021-08-24

**Authors:** Soo Im Chung, Su Noh Ryu, Mi Young Kang

**Affiliations:** 1 Center for Food and Nutritional Genomics Research, Kyungpook National University, Daegu 41566, Korea; zizibe0312@nate.com; 2 Department of Food Science and Nutrition, Kyungpook National University, Daegu 41566, Korea; 3 Department of Agricultural Science, Korea National Open University, Seoul 03087, Korea; ryusn@knou.ac.kr

**Keywords:** rice bran, Superjami, postmenopause, bone metabolism, oxidative stress, aging

## Abstract

Menopause is a matter of concern for women’s health due to a deficiency of female hormones; additionally, reactive oxygen species and aging can cause osteoporosis. Food becomes increasingly interesting as a menopausal woman’s alternative to hormone therapy. The effects of ethanol extracts from dark purple Superjami rice bran on bone metabolism and antioxidant defense systems in menopause-induced animal models were evaluated. Female rats underwent sham surgery or were ovariectomized to induce a menopause-like state. Rats were divided into a sham control group (SHAM), an ovariectomized control group (OVX), and an ovariectomized grou supplemented with Superjami rice bran extract group (OVX-S) and fed for 8 weeks. The OVX groups exhibited significantly more weight gain, amounts of bone turnover biochemical markers (alkaline phosphatase, osteocalcin, and C-terminal telopeptide), bone loss, lipid-peroxidation and oxidative stress than the SHAM group. However, Superjami bran extract added to the diet resulted in a significant reduction in body weight and lipid peroxidation, as well as enhanced bone metabolism and antioxidant enzyme activities, in ovariectomized rats. These results propound that extracts from Superjami rice bran have therapeutic potentiality against bone loss and oxidative stress in menopause-induced states and will be useful in preventing postmenopausal osteoporosis and oxidative damage.

## 1. Introduction

Menopause is a biological aging process in women and is characterized by the permanent discontinuity of menstruation and a natural diminishment of ovarian function [1]. Ovarian hormone deficiency is known to promote metabolic dysfunctions and increase the possibility of obesity, dyslipidemia, diabetes, and cardiovascular disease [2]. Studies have also shown that lack of ovarian hormones, particularly estrogen, could cause oxidative stress and is associated with the pathogenesis of osteoporosis in postmenopausal women [3,4]. Osteoporosis is a chronic bone disease in which the risk of fractures continuously increases due to a decrease in bone mass and degeneration of the microstructure of bone tissue [5]. In postmenopausal women, the rapid decrease of estrogen enhances severe bone loss and bone fragility due to imbalances in bone resorption and formation [3].

Ovariectomy is the surgical removal of both ovaries to mimic the postmenopausal state of women. Surgically menopausal rats have been commonly used as an animal model to study bone diseases caused by estrogen deficiency and were found suitable in assessing potential therapeutic agents for the purpose of prophylaxis and remedy of osteoporosis associated with the post-menopausal state [6]. Past investigations revealed that oxidative stress plays a key role in the pathogenesis of osteoporotic disease, and that an antioxidant diet could prevent bone loss from bone resorption in postmenopausal women as well as in ovariectomized rats [7,8,9,10,11,12]. To prevent oxidative damage and osteoporosis in postmenopausal women, functional foods and dietary supplements with high antioxidant capacity could potentially be useful.

Superjami is a deep purple rice cultivar developed by traditional breeding in Korea, and it is characterized by powerful antioxidant activity because it is particularly rich in cyanidin-3-glucoside as well as anthocyanins [13]. As a result of feeding mice a high-fat diet by supplementing the diet with Superjami rice flour, reductions in body weight, blood glucose, lipid peroxidation, triglyceride, and total cholesterol were reported [14].

Compared to general non-pigmented rice bran, the rice bran of pigmented cultivars has higher antioxidant activity and reducing power due to relatively higher amounts of anthocyanins, phenolics, and γ-oryzanol [15]. In a previous study, it was reported that ethanolic extracts from blackish-purple pigmented rice brans have strong antioxidative, anticarcinogenic, and antimutagenic activities [16]. Superjami is a newly researched and developed colored rice, and previous studies have reported that antioxidant activities and supplementation with germinated rice or rice bran extract improved lipid and glucose metabolism in menopause animal models [17,18,19]. This study attempted to approach the goal of improving women’s health after menopause through the ethanol extract of rice bran, which contains more physiologically active substances than rice endosperm. Hence, the purpose of this study was to investigate the bone metabolism and antioxidant defense system effects of rice bran extract in a menopause-like ovariectomized rat model.

## 2. Materials and Methods

### 2.1. Rice Bran Samples and Chemicals

Superjami rice was bred and cultivated by Korea National Open University (Department of Agronomy, Seoul, Korea), and was harvested in the middle plain area of Korea in September 2015. Rice bran was separated into endosperm and rice bran at a rice processing complex and provided in powder form. All chemicals used in the experiments were of analytical grade and were purchased from Sigma-Aldrich, Inc. (Steinheim, Germany) and Merck KGaA (Darmstadt, Germany).

### 2.2. Preparation of the Bran Extract and Phytochemical Profiles

For the preparation of rice bran extract, Superjami rice bran and 3 times the weight of 70% ethanol were added while shaken continuously at 40 °C for 3 h and subsequently filtered through Whatman paper #1 (GE Healthcare Life Sciences, Pittsburgh, PA, USA). The filtered solution was concentrated on a rotary vacuum rotator (Eyela N-1000, Tokyo, Japan) and used for the animal diet after freeze-drying (Industrial Vacuum Freeze Dryer, SFDTS10K, Samwon ENG, Incheon, Korea). To measure cyanidin-3-glucoside (C3G) contents, 1 g of rice bran was dissolved in 25 mL of 0.1% trifluoroacetic acid (TFA)/95% ethanol and filtered with a 0.45 μm PVDF syringe filter. It was detected at 530 nm with an ODS-5 column (4.6 mm × 250 mm, Nomura Chemical Co., Nagoya, Japan) using an HPLC (Shimadzu Corp., Kyoto, Japan) instrument. The mobile phase was 0.1% TFA-water and 0.1% TFA-acetonitrile, and the flow rate was 1.0 mL/min [20]. Gamma oryzanol was measured with an HPLC apparatus equipped with a C18 column. The mobile phase was methanol, acetonitrile, dichloromethane, and acetic acid (50:44:3:3, *v/v/v/v*), and the flow rate was 1.4 mL/min [21]. For the measurement of ferulic acid, methanol and 5% acetic acid in water was used as the mobile phase, and the flow rate was measured at 1.0 mL/min [22]. The total phenolic content of rice bran extracts was determined using the Folin–Ciocalteu assay. The absorbance value was measured at 735 nm using gallic acid as the standard [23], and the results are presented in Table 1.

### 2.3. Animal Experiment Design

The animals used in this experiment were 12-week-old female Sprague–Dawley rats (Central Laboratory Animal Inc., Seoul, Korea) that underwent bilateral ovarian removal or sham surgery and were fresh watered in an environment controlled with a temperature of 25 ± 1 °C, relative humidity of 50 ± 5%, and a 12 h day-night cycle. During the acclimatization period of one week, the rats were fed a commercial pellet diet and fresh water ad libitum and were divided into one SHAM group (*n* = 10) and two ovariectomized rat groups of OVX (*n* = 10) and OVX-S (*n* = 10). Animals of all groups were fed the AIN93M diet as a basic diet [24], and for the dose setting of the OVX-S group, 1.5% of the Superjami bran extract was added to the diet with reference to the result of a lethal dose over 10 g/kg in a single-dose oral toxicity test of rice bran extract in ICR mice [25,26]. After the 8-week experiment was ended, the rats were fasted for 12 h and sacrificed under anesthesia using carbon dioxide inhalation. Animal blood samples were obtained from the inferior vena cava using heparin-coated syringes, and centrifuged (4 °C, 15 min) plasma was used for the experiment. The protocol of this animal study was approved by the Ethics Committee of Kyungpook National University (no. 2016-0117)

### 2.4. Light Microscopy of Right Femur

Dissection of the right femurs was conducted according to the method of Peled et al. [27], and tissues were stained using hematoxylin and eosin (H&E). The stained femur sections were examined under a light microscope (Nikon Optiphot-2, Nikon Instruments, Tokyo, Japan).

### 2.5. Analysis of Biochemical Markers of Bone Metabolism

Commercial diagnostic kits used to analyze the biochemical indicators of bone metabolism using plasma are as follows: alkaline phosphatase (ALP) and calcium (ALP and Ca assay kits; Cobas, Indianopolis, IN, USA); osteocalcin (Rat Osteocalcin ELISA kit (Immutopics Inc., San Clemente, CA, USA); and C-terminal telopeptides of type I collagen (Rat Laps EIA kit; Immunodiagnostic Systems Inc., Fountain Hills, AZ, USA).

### 2.6. Determination of Lipid Peroxidation

Thiobarbituric acid reactants (TBARS), an indicator of lipid peroxidation of plasma and erythrocytes, were measured according to the method of Ohkawa et al. [28] Briefly, trichloroacetic acid (5%, *v/v*) and 0.06 M thiobarbituric acid were added to 55 μL of plasma and erythrocyte samples and heated at 80 °C for 90 min. After centrifuging the reaction solution, the absorbance of the supernatant was measured at 535 nm.

### 2.7. Measurement of Hepatic and Erythrocyte Antioxidant Enzyme Activities

The enzyme fraction of hepatic tissue was homogenized in a buffer solution containing 0.15 M triethanolamine, 0.25 M EDTA, and 0.005 M dithiothreitol in 0.3 g of liver tissue and centrifuged at 1000× *g* at 4 °C for 15 min. The precipitate obtained by centrifuging the supernatant for 10,000× *g* for 15 min at 4 °C was served as the mitochondrial fraction, and the supernatant was centrifuged again for an hour (105,000× *g*, 4 °C). The supernatant and precipitate separated by centrifugation were used for analysis as cytosol and microsome fractions. Superoxide dismutase (SOD) activity was measured using a spectrophotometer that inhibits auto-oxidation by 50% through a reaction with pyrogallol solution and liver enzymes according to the Marklund and Marklund method [29]. Glutathione peroxidase (GPx) activity was measured after incubation of an assay mixture containing 30 mM glutathione and 6 mM NADPH at 25 °C for 5 min at 340 nm absorbance according to the method of Paglia and Valentine [30]. Catalase (CAT) activity was monitored spectrophotometrically at 240 nm for 5 min for degradation of hydrogen peroxide in reaction solutions containing hepatic enzymes or erythrocytes according to Aebi’s method [31]. Glutathione reductase (GR) activity was measured at 340 nm in the NADPH reduction in the mixed reaction solution according to the method of Mize and Langdon [32]. Paraoxonase (PON) activity was spectroscopically measured for p-nitrophenol produced by reacting the hepatic enzyme source with paraoxon solution at 405 nm at 25 °C for 90 s, referring to the Mackness method [33].

### 2.8. Statistical Analysis

All data related to this experiment were expressed as mean ± standard error (SE), and the data were evaluated using the Statistical Package for Social Sciences software program version 19.0 (SPSS Inc., Chicago, IL, USA). The difference between the means were determined by Tukey’s test; statistical significance was considered at *p* < 0.05.

## 3. Results

### 3.1. Change in Body Weight

Body weight gain was highest in the OVX group (44.6 g) and lowest in the SHAM group (13.8 g) following the end of the experiment period (Table 2). Animal groups showed the highest feed intake in the OVX-S group, but significantly lower weight gain (34.2 g) than the OVX group. Furthermore, food efficiency ratio was lowest in the SHAM group (1.4%) and highest in the OVX group (4.3%).

### 3.2. Biochemical Markers of Bone Metabolism

The amounts of ALP, osteocalcin, and CTx significantly increased in the OVX group relative to the SHAM rats (Table 3), while supplementation of the Superjami rice bran extract conspicuously decreased osteocalcin (14.8 ng/mL) and CTx (6.0 ng/mL) to normal level in ovariectomized rats. The OVX-S rats also showed lower ALP content (47.7 U/L) than the OVX group (64.9 U/L). No significant differences were found in all animal groups for changes in plasma calcium content.

### 3.3. Light Micrographs of Right Femur

**Figure 1:**
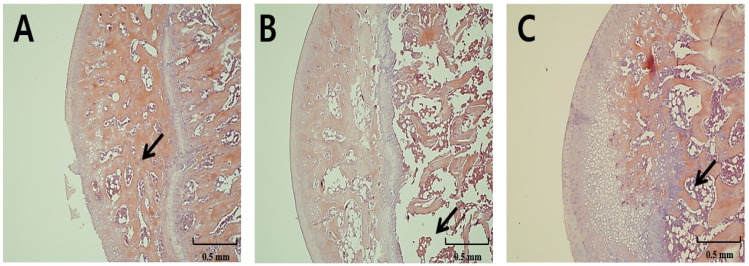
Representative light micrographs of right femur sections of rats (×5). (**A**) SHAM-operated, (**B**) ovariectomized, and (**C**) ovariectomized fed with Superjami rice bran extract. Arrows indicate spaces within the trabeculae that are empty or filled with bone cells.

The SHAM group showed normal bone architecture of the femur with a dense and well-connected trabecular network (Figure 1A). The OVX group, on the other hand, exhibited impaired bone architecture with wide intertrabecular spaces resulting from trabecular bone loss (Figure 1B). However, the OVX-S group showed a denser trabecular network with smaller intertrabecular spaces and decreased bone loss (Figure 1C) relative to the OVX group.

### 3.4. Lipid Peroxidation

As shown in Table 4, the plasma TBARS was significantly higher in the OVX group (8.4 nmol/mL) compared to that of the SHAM mice (3.5 nmol/mL). Likewise, the erythrocyte TBARS considerably increased in OVX mice (20.7 nmol/g Hb) relative to that of the SHAM mice (15.7 nmol/g Hb). However, the bran extract supplementation diet manifestly reduced both plasma (4.4 nmol/mL) and erythrocyte (15.5 nmol/g Hb) TBARS levels in ovariectomized rats.

### 3.5. Activities of Antioxidant Enzymes

Ovariectomy resulted in a significant reduction in the activities of the hepatic and erythrocyte antioxidant enzymes SOD, GPx, CAT, GR, and PON (Table 5). Contrariwise, ovariectomized rats supplemented with Superjami rice bran extract were observed to significantly enhance activities of such antioxidant enzymes.

## 4. Discussion

The promotion of oxidative stress due to estrogen deficiency after menopause is a risk factor for osteoporosis in elderly women [4,9]. The purpose of this study was to investigate the bone metabolism and antioxidant enzyme activity effects of supplementation of the newly bred deep purple Superjami rice bran extract, and to examine the possibility of alternative estrogen replacement therapy for postmenopausal osteoporosis using a menopause-like animal model. As a result of ovariectomy in rats, body weight, lipid peroxidation, and bone loss, as well as the level of biochemical indicators of bone metabolism such as osteocalcin, CTx, and ALP, were significantly increased due to acceleration of bone turnover. Moreover, the antioxidant enzyme activities of SOD, GPx, CAT, GR and PON in hepatic tissue and erythrocytes were significantly lower than in OVX rats compared to SHAM group. A previous study found that ovariectomy in female rats aggravated the bone antioxidant system and significantly increased body weight due to oxidative stress [34,35]. A number of studies have reported increased bone turnover in ovariectomized female rats as indicated by elevated levels of osteocalcin and ALP, which are biochemical indicators of bone formation, as well as CTx, a biochemical indicator of bone resorption. [36,37,38,39]. In adult women, an acceleration in bone turnover, manifested by a 50–100% increase in both bone formation and resorption indicators, occurs after menopause, resulting in a rapid rate of bone loss [40].

The experimental group of ovariectomized r ats fed the diet supplemented with Superjami rice bran extract remarkably reduced the bone turnover, as evidenced by decreased amounts of ALP, osteocalcin, and CTx. In particular, osteocalcin and CTx were maintained at normal levels in the OVX-S group. Furthermore, compared with the OVX group, the OVX-S group significantly decreased TBARS levels in plasma as well as erythrocytes indicating oxidative stress and lipid peroxidation, and the activity of antioxidant enzymes in hepatic and erythrocytes was higher.

The enzymes such as SOD, GPx, CAT, GR and PON that catalyze reactive oxygen species-quenching reactions are part of a very complex system of interactions with antioxidant defenses that regulate oxidative stress. The superoxide radical is converted by the SOD enzyme to hydrogen peroxide, which is detoxified by CAT enzymes in the peroxisome and GPX enzymes in the cytoplasm and mitochondria [41]. The GR enzyme converts the oxidized form of glutathione disulfide to reduced glutathione using NADPH as a coenzyme [42], and the degradation of lipid peroxides and hydrolysis of oxidized phospholipids are accomplished by the PON enzyme [43]. The diminished lipid peroxidation and ameliorated antioxidant enzyme activities observed in the OVX-S group indicate an improvement in the antioxidant defense system of these bran extract-fed ovariectomized animals, making them less easily affected by postmenopausal oxidation damage. Supplemental feeding of Superjami rice flour was found to improve antioxidant enzyme activities and inhibit oxidative stress in hyperlipidemia-induced mice fed a high-fat diet [14].

Pigmented rice varieties like Superjami are high in antioxidants such as tocopherols and phenolic compounds, including anthocyanins. Since most of the bioactive ingredients that can affect metabolism are contained in rice bran with the embryo, it can be used as a functional food and produces a significant effect with only a small amount of food [13,20,44]. Numerous studies have revealed that bran extracts from pigmented rice contain high amounts of phytochemicals that have strong antioxidant capacities [15,23,45,46,47,48].

Ovariectomy has been shown to alter the antioxidant defense system of the cell, resulting in oxidative stress caused by accumulation of reactive oxygen species [35]. Oxidative stress may eventually play a critical role in the pathogenesis of osteoporosis [7]. Grassi et al. reported that bone loss was caused by oxidative stress in estrogen-deficient ovariectomized mice, and that antioxidant treatment could prevent bone loss [49]. Several investigations have also revealed that the approach of a diet based on antioxidants could be beneficial and helpful in preventing and treating postmenopausal osteoporosis [10,11,50]. Therefore, the reduction of lipid peroxidation and the enhancement of antioxidant enzyme activities in OVX-S rats were experimentally verified, and the strong antioxidant ability of Superjami rice bran extract may have contributed to partially improving bone metabolism by reducing bone loss in OVX-S rats. This pigmented rice bran extract can be potentially useful in preventing oxidative damage and bone loss in estrogen-deficient women.

## 5. Conclusions

Dietary replenishment of Superjami rice bran extract noticeably improved bone metabolism and reduced bone loss in ovariectomized rats. It also significantly suppressed oxidative stress and enhanced the activities of antioxidant enzymes. This strong antioxidative effect of the bran extract is possibly responsible for the inhibition of the ovariectomy-induced bone turnover imbalance and impaired bone architecture in the bran extract-fed rats. The results suggest that extracts of rice bran from Superjami could be of practical value in the prevention of postmenopausal osteoporosis and oxidative damage.

## Figures and Tables

**Table 1:** Phytochemical profiles of Superjami rice bran.

Variables	Amount
Cyanidin-3-glucoside (mg/g)	32.14 ± 5.25
γ-Oryzanol (mg/g)	0.36 ± 0.06
Ferulic acid (mg/g)	1.14 ± 0.21
Total phenolic compounds (mg GAE/g)	5.39 ± 0.14

Data are presented as mean ± S.D. (*n* = 3).

**Table 2:** Changes in body weight gain and feed efficiency of Superjami rice bran extract supplementation diet in ovariectomized rats.

Variables	SHAM	OVX	OVX-S
Initial weight (g)	292.49 ± 0.19 ^a^	293.95 ± 2.41 ^a^	294.45 ± 0.21 ^a^
Final weight (g)	306.14 ± 1.09 ^a^	340.67 ± 1.83 ^c^	328.69 ± 1.18 ^b^
Weight gain (g)	13.88 ± 0.98 ^a^	44.60 ± 3.12 ^c^	34.25 ± 0.97 ^b^
Feed intake (g/week)	125.11 ± 1.37 ^a^	130.39 ± 1.41 ^a^	150.50 ± 2.83 ^b^
FER (%)	1.38 ± 0.10 ^a^	4.28 ± 0.30 ^c^	2.84 ± 0.08 ^b^

Data are presented as mean ± S.E. (*n* = 10) and ^abc^ means differ significantly at *p* < 0.05, as common letters in the same row are not shared. SHAM, sham operated rats (AIN93M); OVX, ovariectomized rats (AIN93M); OVX-S, Ovariectomized rats (Superjami rice bran extract in AIN93M); FER, food efficiency ratio = weight gain/feed intake × 100.

**Table 3:** Changes in biochemical markers of bone metabolism levels of Superjami rice bran extract supplementation diet in ovariectomized rats.

Variables	SHAM	OVX	OVX-S
ALP (U/L)	40.01 ± 0.76 ^a^	64.87 ± 4.01 ^c^	47.67 ± 4.33 ^b^
Calcium (mg/dL)	11.09 ± 0.11 ^a^	10.87 ± 0.23 ^a^	11.01 ± 0.41 ^a^
Osteocalcin (ng/mL)	14.69 ± 1.47 ^a^	27.25 ± 2.01 ^b^	14.79 ± 1.80 ^a^
CTx (ng/mL)	5.47 ± 1.07 ^a^	13.87 ± 2.33 ^b^	6.01 ± 1.43 ^a^

Data are presented as mean ± S.E. (*n* = 10) and ^abc^ means differ significantly at *p* < 0.05, as common letters in the same row are not shared. SHAM, sham operated rats (AIN93M); OVX, ovariectomized rats (AIN93M); OVX-S, ovariectomized rats (Superjami rice bran extract in AIN93M); ALP, alkaline phosphatase; CTx, C-terminal telopeptide.

**Table 4:** Changes in plasma and erythrocyte TBARS of Superjami rice bran extract supplementation diet in ovariectomized rats.

Variables	SHAM	OVX	OVX-S
Plasma TBARS (nmol/mL)	3.50 ± 0.06 ^a^	8.45 ± 0.21 ^c^	4.43 ± 0.10 ^b^
Erythrocyte TBARS (nmol/g Hb)	15.70 ± 0.20 ^a^	20.70 ± 0.08 ^b^	15.48 ± 0.42 ^a^

Data are presented as mean ± S.E. (*n* = 10) and ^abc^ means differ significantly at *p* < 0.05, as common letters in the same row are not shared. SHAM, sham operated rats (AIN93M); OVX, ovariectomized rats (AIN93M); OVX-S, ovariectomized rats (Superjami rice bran extract in AIN93M); TBARS, thiobarbituric reactive substances.

**Table 5:** Changes in hepatic and erythrocyte antioxidant enzyme activities of Superjami rice bran extract supplement diet in ovariectomized rats.

Variables	SHAM	OVX	OVX-S
*Hepatic antioxidant enzymes (nmol/min/mg protein)*			
SOD	1.20 ± 0.10 ^c^	0.54 ± 0.02 ^a^	0.67 ± 0.02 ^b^
GPx	3.69 ± 0.02 ^c^	2.56 ± 0.01 ^a^	2.81 ± 0.09 ^b^
CAT	0.77 ± 0.00 ^c^	0.48 ± 0.02 ^a^	0.59 ± 0.01 ^b^
GR	14.01 ± 0.59 ^b^	8.99 ± 0.33 ^a^	12.99 ± 0.48 ^b^
PON	0.19 ± 0.01 ^c^	0.04 ± 0.01 ^a^	0.14 ± 0.00 ^b^
*Erythrocyte antioxidant enzymes (µmol/min/mg/hemoglobin)*			
SOD	1.64 ± 0.04 ^c^	0.89± 0.02 ^a^	1.35 ± 0.05 ^b^
GPx	0.81 ± 0.01 ^c^	0.32 ± 0.02 ^a^	0.56 ± 0.14 ^b^
CAT	0.52 ± 0.03 ^c^	0.24 ± 0.02 ^a^	0.40 ± 0.02 ^b^
GR	0.60 ± 0.01 ^c^	0.26 ± 0.01 ^a^	0.37 ± 0.01 ^b^

Data are presented as mean ± S.E. (*n* = 10) and ^abc^ means differ significantly at *p* < 0.05, as common letters in the same row are not shared. SHAM, sham operated rats (AIN93M); OVX, ovariectomized rats (AIN93M); OVX-S, ovariectomized rats (Superjami rice bran extract in AIN93M); SOD, superoxide dismutase; GPx, glutathione peroxidase; CAT, catalase; GR, glutathione reductase; PON, paraoxonase.

## Data Availability

Data related to the results of this study can be obtained from the corresponding author upon reasonable request.
